# Supplementary material for: An in vitro platform for the enzymatic characterization of the rhomboid protease RHBDL4
Source: J Biol Chem. 2025 Feb 7;301(3):108275. doi: 10.1016/j.jbc.2025.108275 (PMC11929074; doi:10.1016/j.jbc.2025.108275)
Supplement: Supporting information [file mmc1.docx]

**Supporting Information**

**An *in vitro* platform for the enzymatic characterization of the rhomboid protease RHBDL4**

**Satarupa Bhaduri^1^, Mac Kevin E. Braza^2^, Stancho Stanchev^6^, Marina Tauber^5^, Raghad Al-Bawab^1^, Lawrence J. Liu^4^, Diego F. Trujillo^4^, Kristina Solorio-Kirpichyan^1^, Ambuj Srivastava^3^, Javier Sanlley-Hernandez^3^, Anthony J. O’Donoghue^4^, Marius K. Lemberg^5^, Rommie Amaro^3^, Kvido Strisovsky^6^, and Sonya E. Neal^1,6,7,^**^*^

^1^School of Biological Sciences, Department of Cell and Developmental Biology, University of California San Diego, La Jolla, CA 92093, USA

^2^School of Physical Sciences, Department of Chemistry and Biochemistry, University of California San Diego, La Jolla, CA 92093, USA

^3^School of Biological Sciences, Department of Molecular Biology, University of California San Diego, La Jolla, CA 92093, USA

^4^Skaggs School of Pharmacy and Pharmaceutical Sciences*,* University of California, San Diego, La Jolla, CA 92093, USA

^5^Center for Biochemistry, Cologne Excellence Cluster on Cellular Stress Responses in Aging-Associated Diseases (CECAD) and Center for Molecular Medicine Cologne (CMMC), Faculty of Medicine, University of Cologne, Cologne, Germany

^6^Institute of Organic Chemistry and Biochemistry, Academy of Sciences of the Czech Republic, Prague, 160 00, Czech Republic

^7^Howard Hughes Medical Institute, Chevy Chase, MD 20815, USA

Content:

Supplemental Information 1-12

Tables S1 & S2

**
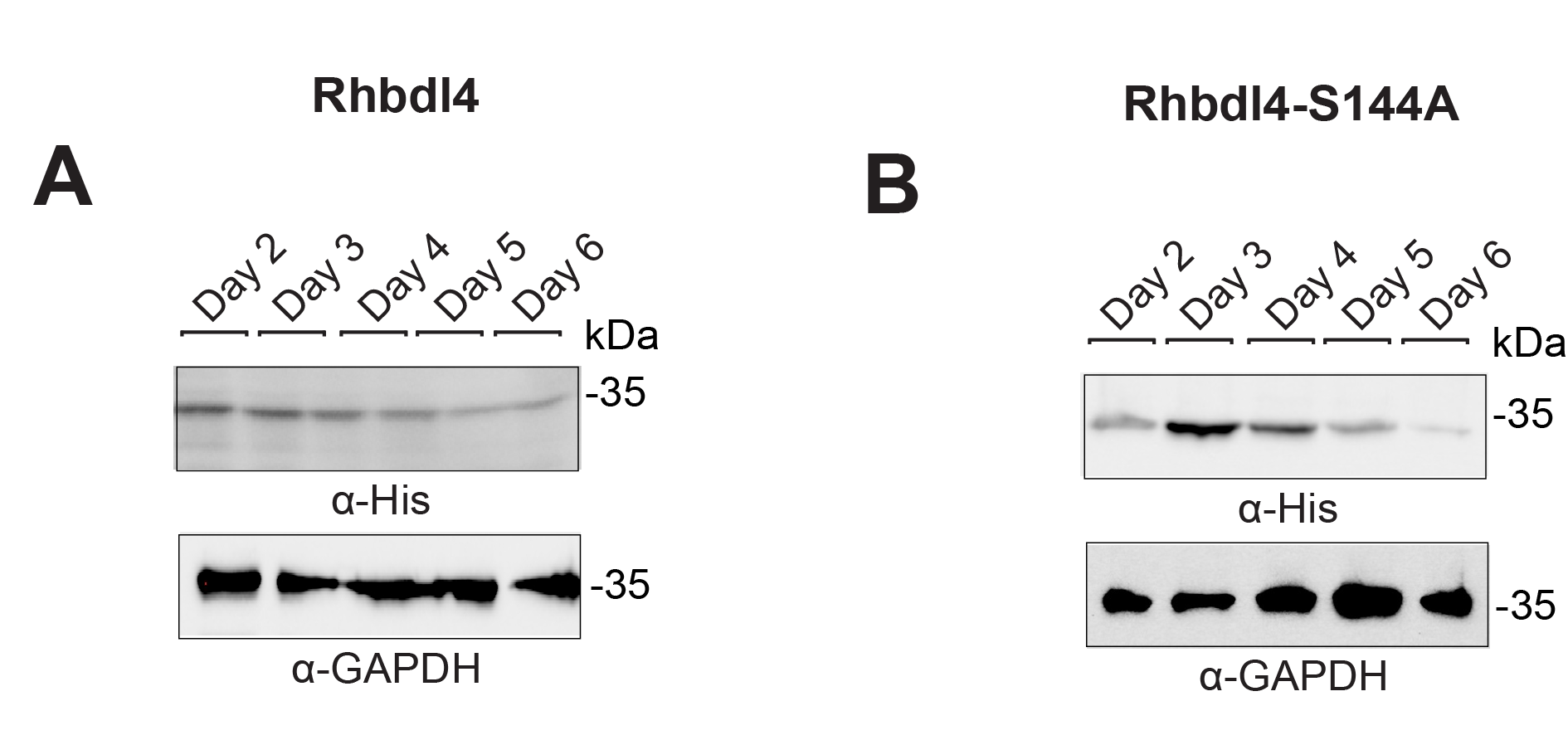
**

**Supplemental Information 1. (A)** Freestyle 293-F suspension cells were transfected with the RHBDL4 plasmid and cells were grown for the indicated number of days. 50 µg of lysate was subjected to immunoblotting for monoclonal α-His antibody and monoclonal α-GAPDH antibody. **(B)** Same as (A) except RHBDL4-S144A was transfected.


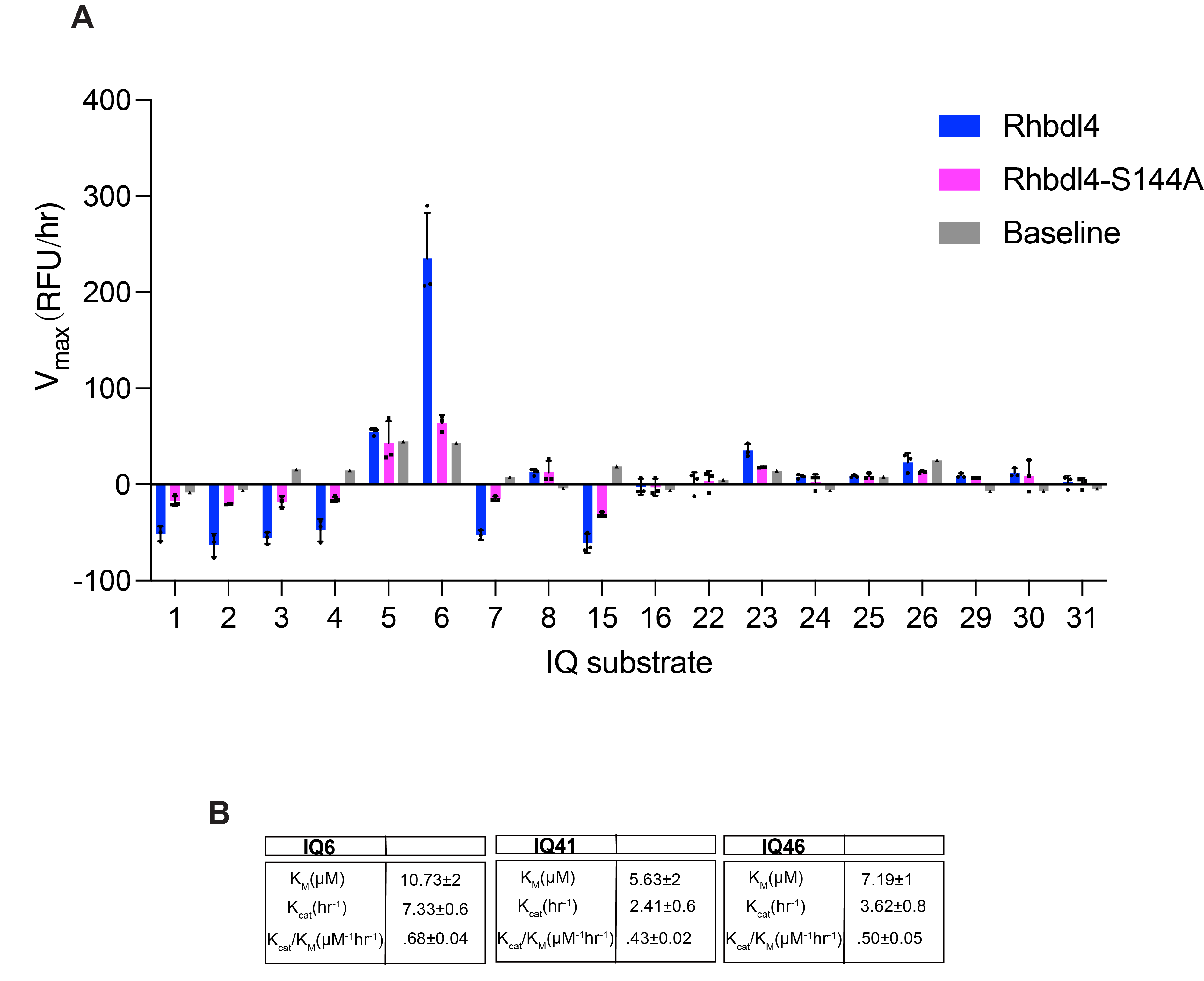


**Supplemental Information 2. (A)** Proteolytic rate of indicated IQ substrates cleaved by RHBDL4. **(B)** Catalytic parameters of IQ6, IQ40, and IQ41 substrate cleavage by RHBDL4.


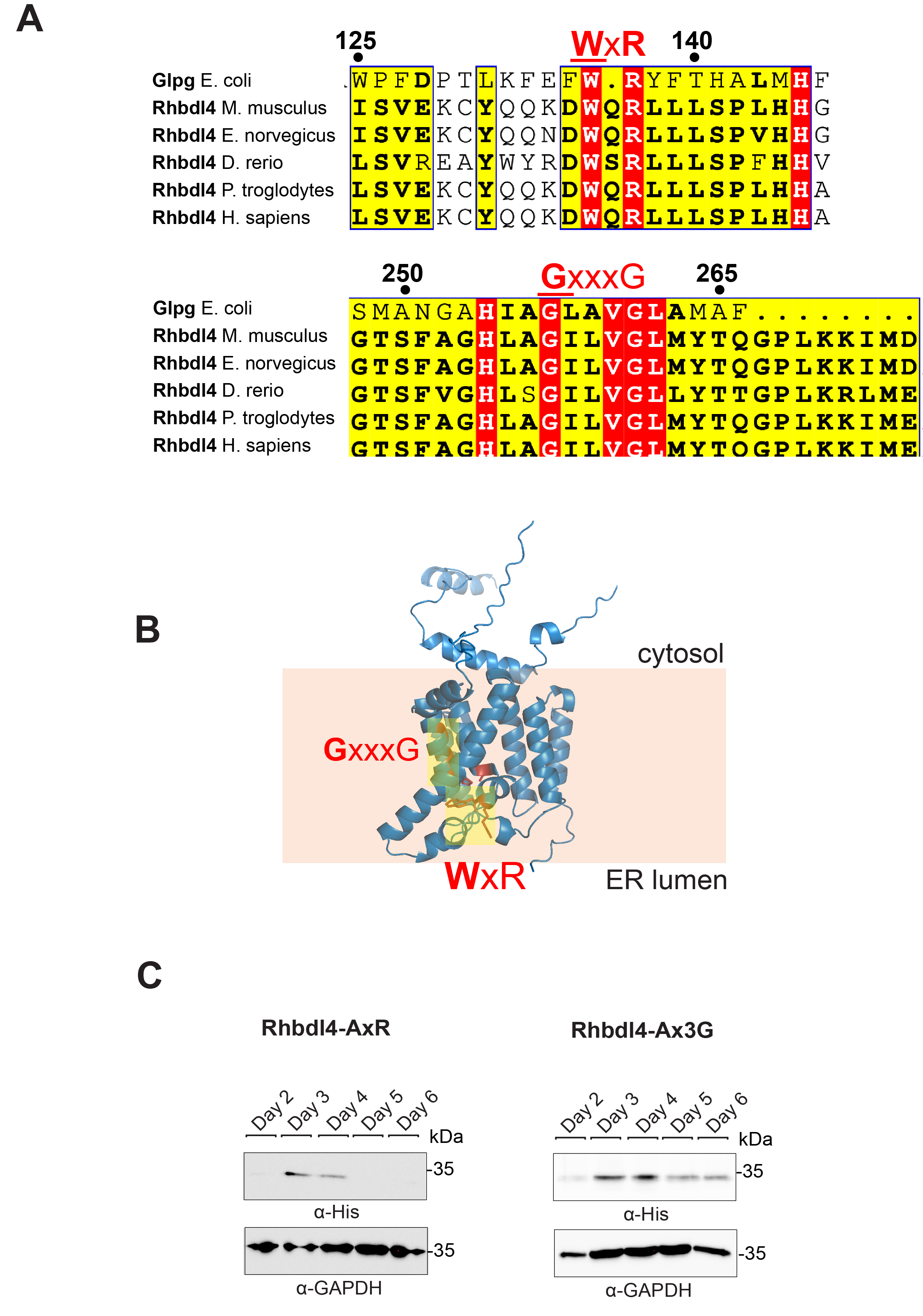


**Supplemental Information 3. (A)** Conservation of RHBDL4. Alignment of RHBDL4 (*H. sapiens, M. musculus, E. norvegicus, D. rerio, P. troglodytes*) with GlpG (*E. coli*). Identical residues are highlighted in red. **(B)** Alphafold model of RHBDL4. Positions of WxR and Gx_3_G motif is highlighted in yellow. **(C)** Freestyle 293-F suspension cells were transfected with RHBDL4-AxR and RHBDL4-Ax3G and cells were grown for the indicated amount of days. 50 µg of lysate was subjected to immunoblotting for RHBDL4 with monoclonal α-His antibody and monoclonal α- GAPDH antibody.

**
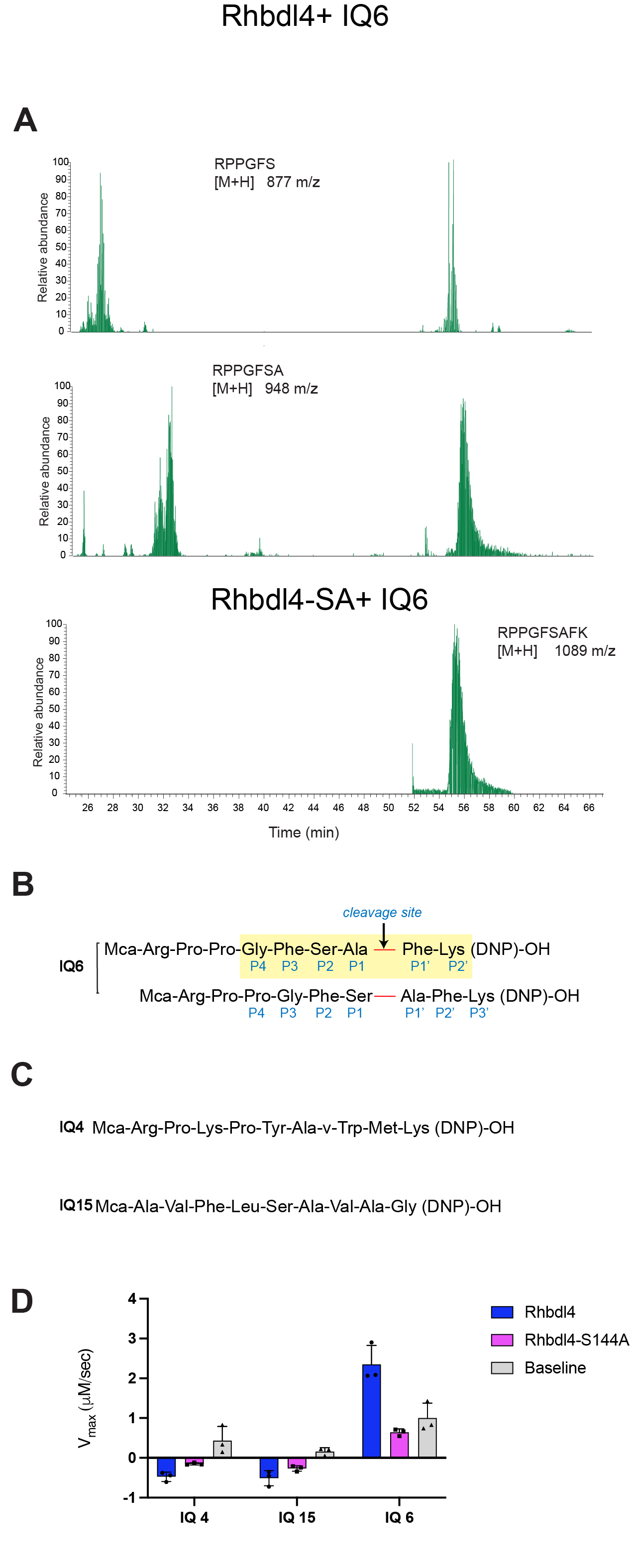
**

**Supplemental Information 4. (A)** Wild-type RHBDL4 and inactive RHBDL4-S144A were incubated with 10 μM of the IQ6 substrate Mca-Arg-Pro-Pro-Gly-Phe-Ser-Ala-Phe-Lys (DNP)-OH for 4 h at room temperature, and the reaction was quenched with 8 M GuHCl, desalted, eluted, and analyzed via tandem mass spectrometry (MS/MS). **(B)** Depiction of IQ6 cleavage sites as determined from tandem mass spectrometry (MS/MS). **(C)** Peptide sequence of GlpG-derived and PARL-derived fluorescent substrates IQ4 and IQ15, respectively. **(D)** Activity profiles of the wild-type RHBDL4 and mutant RHBDL4-S144A, as measured by cleavage of the fluorescent substrates IQ4 and IQ15. Assays was performed in triplicate, and data points are represented by the average ± S.E.M.

**
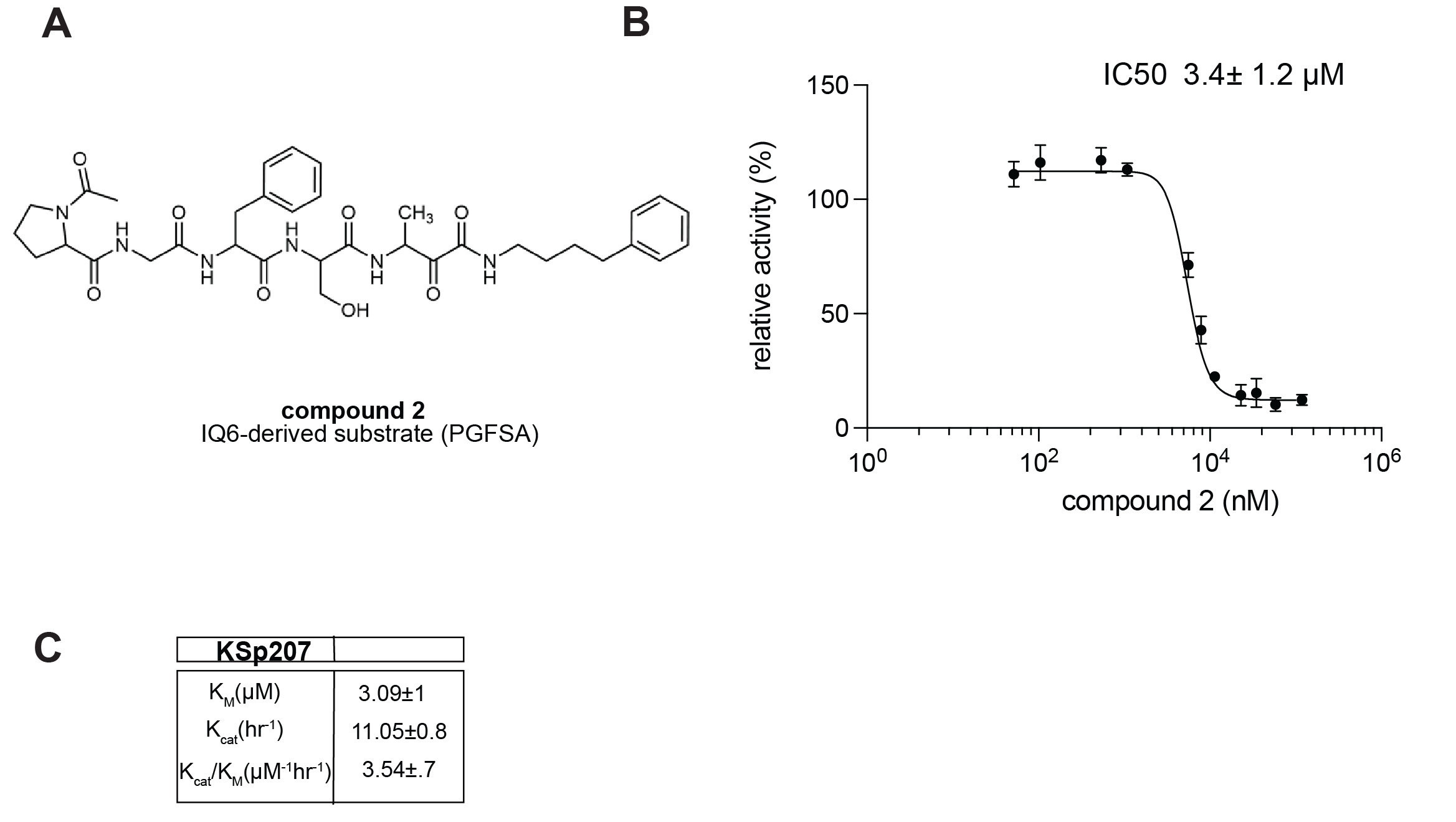
**

**Supplemental Information 5. (A)** Schematic representation of the peptidyl- α-ketoamide inhibitor, compound **2. (B)** Representative inhibition curve derived from measuring rates of RHBDL4 proteolysis of the fluorescent substrate IQ6 with increasing concentrations of compound **2**. **(C)** Catalytic parameters of KSp207 substrate cleavage by RHBDL4.

**Supplemental Information 6. (A)** AlphaFold2 model of RHBDL4 with the highly mobile structure at the N-terminal and C-terminal residues**. (B)** Superimposed structures of the RHBDL4 AlphaFold2 model (without the terminal residues) and top cluster conformation of the apo form RHBDL4 (ER membrane-bound).

**Supplemental Information 7**. RHBDL4 (apo) most populated clusters from all-atom MD simulations. Structures were clustered based from the solvent-accessibility-surface-areas (SASA) values of residues within 10 Angstrom of HIS144 and SER195. Each cluster was represented with the frames that has max SASA values.

**Supplemental Information 8.** RHBDL4 and peptide Ca Root-mean-square deviation from the initial frame of molecular dynamics simulations. Trajectories were aligned. Four replicates for each system were analyzed.

**Supplemental Information 9.** 2D representation of RHBDL4 close interacting residues with the peptide side chains. (A) PGFSA (B) Superimposed PGFSA, QMESA, and MESA. (C) QMESA (D) MESA. All residues that were shared by the three substrate-derived peptides were marked with yellow stars. Ligplot+ was used to render this figure.

**Supplemental Information 10.** Time evolution snapshot of MESA interaction with the RHBDL4 active site.

**Supplemental Figure 11.** Time evolution snapshot of PGFSA interaction with the RHBDL4 active site.

**Supplemental Figure 12.** Time evolution snapshot of QMESA interaction with the RHBDL4 active site.

**Supplemental Table 1:** AutoDock Vina ensemble molecular docking (n=4)

|  | **PGFSA (IQ6)**  mca-RPPGFSAFK(dnp) | **QMESA (Ksp207)**  Ac-QMESA-4mc | **MESA** |
| --- | --- | --- | --- |
| **Binding affinity**(kcal×mol^-1^)* | -6.8 ± 0.5 | -5.2 ± 0.3 | -5.2 ± 0.1 |

**Supplemental Table 2.** Plasmids used in this study

**Supplemental Table 3**. RHBDL4 Close interacting residues with the substrate. Refer to Supplementary Information 9 and main Figure 7 for 3D and 2D renderings.

| **Peptide** | **Close interacting amino acid residues** | |
| --- | --- | --- |
|  | **Hydrophobic interactions** | **Hydrogen bonds** |
| PGFSA | D77 (TM2)  W79 (TM2)  H80 (TM2)  F83 (TM2)  I183 (TM5)  F186 (TM5)  S187 (loop 5) | E179 (TM5) |
| QMESA | D77 (TM2)  W79 (TM2)  H80 (TM2)  F83 (TM2)  I165 (TM5)  E179 (TM5)  A182 (TM5)  I183 (TM5)  S187 (loop 5) | F186 (TM5) |
| MESA | D77 (TM2)  W79 (TM2)  H80 (TM2)  F83 (TM2)  N84 (TM2)  E179 (TM5)  I183 (TM5)  F186 (TM5)  S187 (loop 5) | none |

| **Plasmid #** | **Backbone & Gene** |
| --- | --- |
| pSN182 | pcDNA3.1 HisA RHBDL4 |
| pSN183 | pcDNA3.1 HisA RHBDL4-S144A |
| pSN294 | pcDNA3.1 HisA RHBDL4-AxR |
| pSN295 | pcDNA3.1 HisA RHBDL4-Ax3R |
